# Dental caries status of patients with schizophrenia in Seville, Spain: a case–control study

**DOI:** 10.1186/s13104-016-2368-9

**Published:** 2017-01-18

**Authors:** Eugenio Velasco-Ortega, L. Monsalve-Guil, I. Ortiz-Garcia, A. Jimenez-Guerra, J. Lopez-Lopez, J. J. Segura-Egea

**Affiliations:** 1 Faculty of Dentistry, Dpto. de Estomatología, University of Sevilla, C/Avicena s/n, 41009 Seville, Spain; 2 Faculty of Dentistry, University of Sevilla, Seville, Spain; 3 Faculty of Dentistry, University of Barcelona, Barcelona, Spain

**Keywords:** Dental status, Patients with schizophrenia, Dental caries, Mental disorders, Missing teeth, DMFT

## Abstract

**Background:**

The aim of this study was to assess the dental status (DMFT) in patients with schizophrenia compared with a control group.

**Material:**

In this case–control study, 50 patients with schizophrenia attended in the Psychiatric Unit at the Virgen Macarena University Hospital of Seville were compared with 50 people (without systemic diseases and not taking psychotropic drugs) in a control group attended in the School of Dentistry of Seville. Decayed, missing and filled teeth (DMFT) were assessed according to the World Health Organization WHO criteria.

**Results:**

Patients with schizophrenia showed a decayed teeth (DT) score of 7.26 ± 5.69 compared with 6.50 ± 4.37 for patients the control group. These differences were significant and suggest that dental caries are most prevalent in patients with schizophrenia. People who smoked showed significantly higher DT scores in both groups. Among patients with schizophrenia, smokers scored 9.34 ± 5.42 compared with 4.38 ± 4.82 for non-smokers. Among the healthy controls, smokers scored 6.88 ± 4.85 compared with 6.12 ± 3.85 for non-smokers (p < 0.05). Patients with schizophrenia showed a missing teeth (MT) score of 9.10 ± 8.56 compared with 5.38 ± 5.14 in control patients. MT scores increased significantly with age and with smoking in both groups of patients (p < 0.05). Patients with schizophrenia showed a filled teeth (FT) score of 1.38 ± 2.70 compared with 2.34 ± 3.48 in control patients. FT differences in gender and smoking habits between patients with schizophrenia and healthy control subjects were statistically significant (p < 0.05). This data, along with the DT scores, suggests that patients with schizophrenia have extensive untreated dental disease.

**Conclusions:**

Patients with schizophrenia constitute a high risk population for dental health. This group showed a greater prevalence of decayed and missing teeth and more extensive treatment needs.

## Background

The prevalence of mental disorders is high and causes profound health, family, social, cultural and economic problems worldwide. Schizophrenia is the most complex psychiatric disorder that affects mankind [1]. Epidemiologically, the incidence of mental disorders in the general population is significant (4.5 per 1000 persons), and is higher in urban settings in developed nations [2, 3]. The majority of people with schizophrenia suffer a variety of medical comorbidities (such as emphysema, obesity, lung cancer, and cardiac disease) as well as an increased risk of death [2–4].

Several studies of the oral health of psychiatric patients in different countries have showed that mental illnesses and the psychotropic medications used to treat them can increase the prevalence and the severity of dental diseases (e.g., caries) [5–20]. Schizophrenia should be considered a risk factor for poor oral health because patients with schizophrenia are likely to face a unique set of factors that lead to the development of advanced oral diseases—and to receive less oral health care [21–28].

Psychotropic medications may cause an important salivary gland hypofunction. In fact, decreased salivation is the oral adverse effect of such medications most frequently reported by patients and clinicians. Phenothiazines, atypical antipsychotics, benzodiazepines and antiparkinsonians drugs have been shown to cause salivary hypofunction and xerostomia in patients with schizophrenia [3, 6, 12, 22, 24]. Importantly, patients with schizophrenia who experience reduced salivary flow have an increased incidence of dental caries [26, 27]. Hyposalivation causes the intensification of cariogenic microflora because of the adverse changes in the oral environment and increases the rapid progression of tooth decay [6, 10, 12, 14, 16, 22]. Another factor that contributes to increased incidence of dental caries in patients with schizophrenia is higher sugar intake. Snacking frequency is associated with a higher prevalence of dental caries and plaque index, and this frequency is higher in this kind of patients than in general population [6, 17, 28].

Chronic psychiatric patients with schizophrenia have shown a lack of motivation in the maintenance of dental hygiene. Negative symptoms and personality disorders of mental illness may be responsible for poor tooth brushing habits [11, 23, 24, 28]. Almost all patients with schizophrenia have large amounts of plaque and calculus and high bleeding indices [6, 9, 10, 13, 17, 18]. Smoking habits are highly problematic in patients with schizophrenia and contribute to worse dental hygiene and increased loss of periodontal attachment [16–19, 24, 25]. Tobacco use affects the morphology and motility of human gingival and periodontal ligament fibroblasts, which facilitate the development and progression of periodontal disease. In addition, tobacco use is a harmful habit that is associated with other non-hygienic habits. Moreover, water pipe smoking has been linked with pathological conditions such as greater inflammation, *candidiasis*, periodontitis, dry socket, elevated blood chromium and nickel levels, premalignant lesions, oral cancer, oesophageal squamous cell carcinoma, attic retraction, oedema in the vocal cords, and lower habitual vocal pitch and voice turbulence index [29, 30].

People with schizophrenia receive suboptimal oral health care and visit dentists less frequently than the general population of the same community [25, 26]. Several studies of the dental health of these patients have shown that they have extensive dental needs, which has a negative impact on their quality of life [5, 6, 12, 20, 25, 27]. Long-term hospitalisation may result in increased oral diseases, especially dental caries and periodontal disease, because preventive dental care is not an integral part of psychiatric care [10, 11, 14–16, 23, 27].

While clinical investigations on the dental health of patients with schizophrenia have increased in recent decades, no specific studies have been conducted to assess the frequency and severity of tooth decay in this group of patients in relation to the rest of the population. The aim of this study was to compare the DMFT scores of patients with schizophrenia to those of healthy control patients, and to study the possible relationships between dental status and different variables.

## Methods

The study was conducted with two groups of patients: patients with schizophrenia and dental patients without mental disorders (the control group). The study was conducted from February 2012 to March 2013. Patients with schizophrenia were selected using a random stratified method according to age and gender, from a total population of 400 patients attending the Psychiatry Department at the University Hospital Virgen Macarena in the Faculty of Medicine of Seville. Diagnosis of schizophrenia was established according the international criteria of the American Psychiatric Association [31].

The control group was selected from among healthy subjects seeking dental treatment at the Faculty of Dentistry of Seville. Using the medical surveys of the Faculty, we selected only people without any system health problems (ASA I). Patients taking any medication for any systemic disease or condition in the 6 months prior to the study were excluded. The selection of control subjects was conducted in consideration of the age and gender of the patients recruited previously (case–control study, one control for each case). The Research Ethics Committee of the University of Seville approved the study, and all participants gave their written informed consent.

Patients with schizophrenia, or their legal guardians, gave consent to collect the necessary data in this study, always preserving the anonymity of the patients. Therefore, demographic data for the patients with schizophrenia were obtained from their medical records, as collected by hospital staff; similar information was garnered directly from the control patients. The demographic variables include age and gender, and also habit smoking, diagnostic psychiatric and taking of drugs.

Dental examinations took place in the Faculty of Dentistry of Seville for healthy control patients, and in the Psychiatry Department of University Hospital Virgen Macarena of Seville for patients with schizophrenia. Each patient’s dental status was determined according to criteria set by the World Health Organization [32]. The decayed, missing and filled teeth (DMFT) score was used to assess the dental status of patients. DMFT were assessed in both groups of patients, according to demographic (age and sex) and clinical (smoking and psychotropic medications) data. All of the dental analyses were performed by the same two operators (EV-O and LM-G), and the explorer was always the same (EV-O).

Statistical analyses were carried out using SPSS Version 17.0 (SPSS Inc., Chicago, IL, USA). Chi square test was used to analyse the differences between proportions. Analysis of variance (ANOVA) was used to detect differences between means. Statistical significance was established at p < 0.05. A Kolmogorov–Smirnov test was used to ensure the normality of data. Sample size was selected to identify changes of an amplitude of 1.5 in a quantitative variable (DMFT score), with a power of test of 80%, an error alpha of 5%, and a common standard deviation of 2.5.

## Results

Fifty patients with schizophrenia (39 males and 11 females) with a mean age of 39.9 years (range: 18–64 years) were examined, along with 50 healthy control subjects (33 males and 17 females) with a mean age of 39.5 years (range: 20–67 years) (Table 1). Differences in age (ANOVA; p = 0.8542) and gender (Chi square test; p = 0.18145) between the two groups were not statistically significant.

**Table 1:** Distribution of patients

	Patients with schizophrenia		Control	
	n	%	n	%
Age (years)				
<35	13	26	19	38
36–45	25	50	17	34
>46	12	24	14	28
Gender				
Males	39	78	33	66
Females	11	22	17	34
Smoking				
Smokers	29	58	26	52
Non-smokers	21	42	24	48
Total	50	100	50	100

All patients with schizophrenia were treated with two or more psychotropic drugs (3.9 ± 2.2). Fifty-eight percent of patients were taking more than four psychotropic medications. Phenothiazines (72%), atypical antipsychotics (60%), benzodiazepines (50%) and antiparkinsonians (50%) were the most common psychotropic drugs used. Twenty-nine of the patients with schizophrenia (58%) and 26 members of the control group (52%) were smokers (Table 1). Differences in the percentage of smokers in the two groups were not statistically significant (Chi square; p = 0.12000).

Patients with schizophrenia expressed a mean number of decayed teeth (DT) of 7.26 ± 5.69. Healthy controls showed a DT of 6.50 ± 4.37 (Table 2). These differences were significant (p = 0.0217). Among patients with schizophrenia, DT scores were highest in 36- to 45-year-old patients (8.28 ± 5.22). These differences were not significant (p = 0.3368). DT scores among healthy control subjects were highest in patients younger than 35 years old (7.84 ± 4.69), but did not show significant differences (p = 0.0610) (Table 2).

**Table 2:** Distribution of dental status

	Patients with schizophrenia				Control			
	D	M	F	DMFT	D	M	F	DMFT
Age (years)								
<35	5.38 ± 3.81	3.92 ± 3.25^d^	2.61 ± 3.73	11.92 ± 3.70^g^	7.84 ± 4.69	2.42 ± 2.41^i^	1.31 ± 2.08	11.57 ± 5.18^j^
36–45	8.28 ± 5.22	9.00 ± 7.34^d^	0.88 ± 2.02	18.16 ± 8.17^g^	6.88 ± 4.18	5.58 ± 3.58^i^	3.00 ± 4.10	15.47 ± 3.57^j^
>46	7.16 ± 7.89	14.91 ± 11.36^d^	1.08 ± 2.46	23.16 ± 9.59^g^	4.28 ± 3.47	9.14 ± 6.90^i^	2.92 ± 4.06	16.35 ± 6.49^j^
Gender								
Males	7.69 ± 5.60	8.92 ± 8.30	0.97 ± 2.24^e^	17.58 ± 9.17	6.18 ± 4.35	5.48 ± 5.79	2.21 ± 3.78	13.87 ± 4.85
Females	5.72 ± 6.01	9.72 ± 9.85	2.81 ± 3.73^e^	18.27 ± 6.11	7.17 ± 4.47	5.17 ± 3.69	2.58 ± 2.91	14.94 ± 4.85
Smoking								
Smokers	9.34 ± 5.42^c^	10.27 ± 8.48	0.68 ± 1.73^f^	20.31 ± 7.81^h^	6.88 ± 4.85	5.92 ± 6.01	2.07 ± 3.01	14.88 ± 6.40
Non-smokers	4.38 ± 4.82^c^	7.47 ± 8.62	2.33 ±3.48^f^	14.19 ± 8.40^h^	6.12 ± 3.85	4.79 ± 4.04	2.62 ± 3.97	13.54 ± 4.24
Total	7.26 ± 5.69^a^	9.10 ± 8.56^b^	1.38 ± 2.70	17.74 ± 8.54	6.50 ± 4.37^a^	5.38 ± 5.14^b^	2.34 ± 3.48	14.24 ± 4.85

The data with the same letter show statistical differences (p<0.05)

^a–j^ Statistically significant differences

Among patients with schizophrenia, males showed higher DT scores (7.69 ± 5.60) compared with females (5.72 ± 6.01). These differences were not significant (p = 0.3169). Among the healthy control subjects, males showed lower DT scores (6.18 ± 4.35) than females (7.17 ± 4.47). These differences were not significant (p = 0.4522) (Table 2).

In the group of patients with schizophrenia, those who smoked showed higher DT scores (9.34 ± 5.42) compared with non-smokers (4.38 ± 4.82). These differences were significant (p = 0.0016). In the healthy control group, smokers showed higher DT scores (6.88 ± 4.85) compared with non-smokers (6.12 ± 3.85). These differences were not significant (p = 0.5452) (Table 2).

All 50 of the patients with schizophrenia (100%) showed at least one missing tooth. Two patients (4%) were totally edentulous. The mean number of missing teeth (MT) was 9.10 ± 8.56. Forty-three healthy control patients (86%) showed at least one missing tooth, with an MT score of 5.38 ± 5.14. These differences were significant (p = 0.0098) (Table 2).

MT scores increased significantly with age among the patients with schizophrenia (p = 0.0039) and those in the control group (p = 0.0004). Among the patients with schizophrenia, females showed higher MT scores (9.72 ± 9.85) compared with males (8.92 ± 8.30). These differences were not significant (p = 0.7865). In the healthy control group, males showed higher MT scores (5.48 ± 5.79) than females (5.17 ± 3.69). These differences were not significant (p = 0.8432) (Table 2).

In the group of patients with schizophrenia, smokers showed higher MT scores (10.27 ± 8.48) compared with non-smokers (7.47 ± 8.62). These differences were not significant (p = 0.2583). Among the healthy controls, smokers showed higher MT scores (5.92 ± 6.01) compared with non-smokers (4.79 ± 4.04). These differences were not significant (p = 0.4427) (Table 2).

Fourteen patients with schizophrenia (28%) showed at least one filled tooth. The mean number of filled teeth (FT) among this group was 1.38 ± 2.70. Twenty-five healthy control patients (50%) showed at least one filled tooth, with an average FT score of 2.34 ± 3.48. These differences were not significant (p = 0.1274). Differences in the number of FT among patients with schizophrenia with respect to gender (p = 0.0451) and smoking (p = 0.0328) were statistically significant (Table 2).

The mean DMFT of the patients with schizophrenia and the healthy control group were 17.74 ± 8.54 and 14.24 ± 4.85, respectively. DMFT scores increased significantly with age for both the patients with schizophrenia (p = 0.0027) and the controls (p = 0.0204). DMFT differences with respect to smoking (p = 0.0109) among patients with schizophrenia were statistically significant (Table 2).

## Discussion

Before discussing our results, we must assume that our study presents some limitations. The selection of subjects without mental disorders for the control group was based on a medical questionnaire and the anamnesis of the patient. This method carries the risks that a questionnaire could be inaccurate or an individual patient could be dishonest. However, the comparison between the two groups seems to indicate that the groups are comparable. The smoking rate among study subjects was high, and this could affect the extrapolation of our results to other populations, especially outside of Spain.

Accepting the above, the clinical findings of this study demonstrate differences in the prevalence and distribution of decayed teeth (DT), missing teeth (MT), filled teeth (FT) and DMFT between patients with schizophrenia and healthy control subjects. Decayed and missing teeth were significantly more prevalent in patients with schizophrenia, suggesting a lack of restorative care with extensive unmet dental treatment needs [6, 16, 23, 24, 27, 28]. In the present study, the DMFT score of the group with schizophrenia was comparable to scores found by other studies concerning non-institutionalised patients with schizophrenia in Mediterranean countries with similar demographic variables [23, 24]. A previous Greek study in outpatients with schizophrenia showed a lower DMFT (16.08) with a DT score of 2.26, MT score of 9.68 and FT score of 2.92 [23]. Also, a recent Spanish study reported a lower DMFT score (13.51) with a DT score of 4.39, MT score of 5.66, and FT score of 3.53 among psychiatric patients with schizophrenia [24]. The main differences may be explained because the combination of decayed teeth and missing teeth in the present study resulted in high DMFT values. The FT scores showed a lower number of filled teeth. The present study reported an MT score of 9.10 ± 8.56 missing teeth among patients with schizophrenia, and 40% of these patients were missing 9-32 teeth. In contrast, the control group had a lower MT score (5.38 ± 5.14) with 22% of subjects missing 9–32 teeth.

The present study indicated significant differences in the number and distribution of decayed teeth. DT scores were higher in patients with schizophrenia compared with controls. Overall, 44% of patients with schizophrenia and 37% of control subjects showed 8 or more decayed teeth. Mental disorders such as schizophrenia may be a risk factor for dental health [3, 4]. A relationship between negative psychopathological states and higher prevalence of decayed teeth has been evidenced [23, 24]. Negative symptoms of schizophrenia impair the ability of patients to exercise preventive oral hygiene [23]. Moreover, chronically hospitalised patients tend to have more severe negative symptoms than patients who live in the community [24]. In fact, long-term hospitalisation causes declines in self- and oral hygiene and dental care, resulting in increased dental caries [10–16]. DMFT scores were higher among hospitalised patients with schizophrenia, but these patients were also older (range of age of 48–58 years) [10, 12, 14–17, 27]. Several studies reporting on hospitalised chronic psychiatric patients with schizophrenia reported high DT scores and MT scores and lower FT scores [12–16]. In addition, one study showed a lower mean DMFT score of 16.08 in outpatients with schizophrenia compared with patients with schizophrenia who had been hospitalised for up to 10 years (18.50) and those hospitalised for more than 10 years (27.17) [23].

The majority of patients with schizophrenia take psychotropic medications on a regular basis. Relevant medications include conventional and atypical antipsychotics, benzodiazepines and antiparkinsonians [14–16]. Psychotropic medications can contribute to dental caries in patients with schizophrenia, as many of them cause dry mouth due to reduced salivary flow [3, 6, 12, 16]. An American study showed that 99% of smooth surface caries (coronal and root) was associated with low salivary flow [6]. Root surface caries, generally associated with older populations, were notably high in a group of young psychiatric patients with schizophrenia [6]. A Turkish study showed a 39.9% correlation of oral dryness in chronic psychiatric patients with the administration of these medications [mean: 2.3 (number of medications per patient)]. Patients with schizophrenia had the highest incidence of symptoms of oral dryness [16]. These findings were confirmed by another study in Israel, where 94% of inpatient study subjects were taking psychotropic medications [mean 2 (number of medications per patient)] and 22% reported experiencing dry mouth [14].

The results showed that the prevalence of missing teeth increased significantly with age in both groups. For all age groups, the mean number of missing teeth (MT) was higher in patients with schizophrenia compared with the control subjects. The schizophrenic group showed a significant age-related increase in DMFT with a corresponding significant increase in the MT score and decrease in the DT component. The mean DMFT score in the oldest age group (over 46 years) was approximately twice that of the youngest age group (up to 35 years). These findings are consistent with those seen in similar psychiatric groups demonstrating that age is an important determinant of poor oral health [12, 14, 15, 17, 24, 28]. Among the patients with schizophrenia, the carious disease process seems to progress over time, and teeth are lost rather than being restored. This is indicated by the increases in MT scores over time [12, 14]. The high prevalence of missing teeth with age may be interpreted to suggest that patients with schizophrenia who seek dental care often have their teeth extracted rather than seeking restorative treatment, probably because of the complexity of their dental care needs [10–14].

In the present study, the gender distribution of the patients with schizophrenia (39 males and 11 females) and the control subjects (33 males and 17 females) were similar. In addition, the DMFT scores in this study did not vary greatly between males and females. These results are confirmed by two similar comparative studies of oral health in patients with schizophrenia, also in Spain, which showed that gender did not affect the dental parameters measured in both study and control groups [20, 24]. A study in Taiwan reported the results of DMFT for males and females among inpatients with schizophrenia (13.93 vs. 13.98) and general patients (7.1 vs. 9.73) with no significant differences [27]. Where differences have been reported, men have worse dental disease than women [14–17, 20]. In patients with schizophrenia, males tend to show higher numbers of carious teeth and root remnants caused by negligence of dental care compared with females [14, 16].

Most people suffering schizophrenia smoke heavily. Smoking is associated with medical and dental problems in patients with schizophrenia. A comparative study showed a significantly higher proportion of smokers in patients with schizophrenia (71%) than controls (39%) (24). A similar trend in smoking prevalence is observed in older patients with schizophrenia (52%) compared with older controls (23%) [25]. However, in the present study the prevalence of smoking in the schizophrenia group (56%) was similar to that in the control group (52%). Smoking can contribute to poor dental health as it is associated with increased periodontal disease and higher rates of tooth loss. Smoking has been related to higher DMFT scores in patients with schizophrenia [28]. Higher numbers of missing and decayed teeth have been reported in smokers compared to non-smokers among patients with schizophrenia [24]. These results are confirmed by the present study: analyses of each parameter individually (D, M and F teeth) revealed that smoking affected the number of missing and decayed teeth. The D and M values were higher among smokers compared with non-smokers. Moreover, the mean DT and MT scores were higher in smokers with schizophrenia than in the smokers of the control group.

The overall percentage of smokers in Spain is 40% (one of the highest rates in Europe) [33]. In our sample, the percentage ranged from 50 to 60% in both groups. We do not think this invalidates our conclusions, although they may not be accurately extrapolated to populations with others percentages of smokers, especially given the effect that smoking can have on other habits and behaviours.

Another important finding from this study is the low number of filled teeth (FT). Among patients with schizophrenia the number was 1.38 ± 2.70, while in the control group it was 2.34 ± 3.48. These clinical findings are consistent with those of other studies, which have demonstrated that patients with schizophrenia are in need of preventive and restorative dental treatment [12, 17, 20, 23]. Illness chronicity and psychopathology severity may explain why patients with schizophrenia visit dentists much less frequently than the general population [26]. Negative symptomatology may cause patients to visit the dentist only when they have severe dental problems that are difficult to treat [23]. Schizophrenia may severely interfere with patients seeking dental treatment, delaying restorative treatment until tooth loss is inevitable [17]. Moreover, dental diseases often represent a lower priority in the overall health care of patients with schizophrenia, but these have a negative impact on the general quality of life [27]. The use of a multidisciplinary model involving mental and oral health is fundamental to the development of dental programs. These programmes must educate and sensitise psychiatrists and psychiatric nurses to dental health. Access to skilled dental care can improve most dental problems for patients with schizophrenia [23].

## Conclusion

The clinical findings of this study demonstrate that patients with schizophrenia should be considered a high-risk group for dental disease. Patients with schizophrenia showed worse dental health than the general population. This study found that age and smoking in psychiatric patients with schizophrenia are determinants for dental caries. Moreover, this study showed a significant rate of missing teeth and confirmed the great need for preventive and restorative dental treatment in this special population.

## Abbreviations

D: decayed
M: missing
F: filled
DMF: decayed, missing, filled

## References

[1] Thornicroft G, Tansella M (2004). Components of a modern mental health service: a pragmatic balance of community and hospital care. Overview of systematic evidence. Br J Psychiatr..

[2] McGrath J, Saha S, Chant D, Welhan J (2008). Schizophrenia: a concise overview of incidence, prevalence, and mortality. Epidemiol Rev.

[3] Friedlander AH, Marder SR (2002). The psychopatology, medical management and dental implications of schizophrenia. J Am Dent Assoc.

[4] McCreadi RG, Stevens H, Hederson J, Hall D, McCaul R, Filik R (2004). The dental health of people with schizophrenia. Acta Psychiatr Scand.

[5] Barnes GP, Allen EP, Parker WA, Lyon TC, Armentrout W, Cole JS (1988). Dental treatment needs among hospitalized adult mental patients. Spec Care Dent.

[6] Stiefel DJ, Truelove EL, Menard TW, Anderson VK, Doyle PE, Mandel LS (1990). A comparison of the oral health of persons with and without chronic mental illness in community settings. Spec Care Dent.

[7] Hede B, Petersen PE (1992). Self-assessment of dental health among Danish noninstitutionalized psychiatric patients. Spec Care Dent.

[8] Ter Horst G (1992). Dental care in psychiatric hospital in the Nederlands. Spec Care Dentist.

[9] Vigild M, Brinck JJ, Christensen J (1993). Oral health and treatment needs among patients in psychiatric institutions for the elderly. Commun Dent Oral Epidemiol.

[10] Angelillo IF, Nobile CGA, Pavia M, De Fazio P, Puca M, Amati A (1995). Dental health and treatment needs in institutionalized psychiatric patients in Italy. Commnity Dent Oral Epidemiol.

[11] Hede B (1995). Oral health status in Danish hospitalized psychiatric patients. Community Dent Oral Epidemiol.

[12] Velasco E, Machuca G, Martinez-Sahuquillo A, Ríos V, Lacalle JR, Bullón P (1997). Dental health among institutionalized psychiatric patients in Spain. Spec Care Dent.

[13] Velasco E, Bullón P (1999). Periodontal status and treatment needs among Spanish hospitalized psychiatric patients. Spec Care Dent.

[14] Ramon T, Grinshpoon A, Zusman SP, Weizman A (2003). Oral health and treatment needs of institutionalized chronic psychiatry patients in Israel. Eur Psychiatr..

[15] Chu KY, Yang NP, Chou P, Chiu HJ, Chi LY (2010). Factors associated with dental caries among institutionalized residents win Taiwan: a cross-sectional study. BMC Public Health..

[16] Gurbuz O, Alatas G, Kurt E (2010). H. Issever H, Dogan F. Oral health and treatment needs of institutionalized chronic psychiatric patients in Istambul, Turkey. Community Dent Health.

[17] Jovanovic S, Milovanovic SD, Gajic I, Mandic J, Latas M, Jankovic L (2010). Oral health status of psychiatric in-patients in Serbia and implications for their dental care. Croat Med J.

[18] Adeniyi AA, Ola BA, Edeh CE, Ogunbanjo BO, Adewuya AO (2011). Dental status of patients with mental disorders in a Nigerian teaching hospital: a preliminary study. Spec Care Dent.

[19] Gopalakrisshnapillai AC, Iyer RR, Kalantharakath T (2012). Prevalence of periodontal disease among inpatients in a psychiatric hospital in India. Spec Care Dent.

[20] Velasco-Ortega E, Segura-Egea JJ, Córdoba-Arenas J, Jiménez-Guerra A, Monsalve-Guil L, López-López J (2013). A comparison of dental status and treatment needs of older adults with and without chronic mental illness in Sevilla, Spain. Med Oral Patol Oral Cir Bucal.

[21] Friedlander AH, Brill NQ (1986). The dental management of patient with schizophrenia. Spec Care Dent.

[22] Friedlander AH, Liberman RP (1991). Oral health care for the patient with schizophrenia. Spec Care Dent.

[23] Thomas A, Lavrentzou E, Karouzos C, Kontis C (1996). Factors which influence the oral condition of chronic schizophrenia patients. Spec Care Dent.

[24] Arnaiz A, Zumárraga M, Díez-Altuna I, Uriarte JJ, Moro J, Pérez-Ansorena MA (2011). Oral health and the symptoms of schizophrenia. Psychiatr Res..

[25] Janardhanam T, Cohen CI, Kim S, Rizvi BF (2011). Dental care and associated factors among older adults with schizophrenia. J Am Dent Assoc.

[26] Nielsen J, Munk-Jorgersen P, Skadhele S, Correll CU (2011). Determinants of poor dental care in patients with schizophrenia: a historical, prospective database study. J Clin Psychiatr..

[27] Chu KY, Yang NP, Chou P, Chiu HJ, Chi LY (2012). Comparison of oral health between inpatients with schizophrenia and disabled people or the general population. J Formos Med Assoc.

[28] Tani H, Uchida H, Suzuki T, Shibuya Y, Shimanuki H, Watanabe K (2012). Dental conditions in inpatients with schizophrenia: a large-scale multi-site survey. BMC Oral Health..

[29] Munshi T, Heckman CJ, Darlow S (2015). Association between tobacco water pipe smoking and head and neck conditions: a systematic review. J Am Dent Assoc.

[30] Wyganowska-Swiatkowska M, Nohawica MM (2015). Effect of tobacco smoking on human gingival and periodontal fibroblasts. A systematic review of literature. Przegl Lek.

[31] American Psychiatric Association (2000). Diagnostic and statistical manual of mental disorders (DSM-IV-TR).

[32] World Health Organization (1997). Oral Health surveys-Basic Methods.

[33] European Commission—Press release. World No Tobacco Day: Eurobarometer reveals that tobacco use is down by 2 percentage points in the EU since 2012, but 26% of Europeans are still smokers. Brussels, 29 May 2015 [http://ec.europa.eu/health/tobacco/docs/2015_infograph_en.pdf].

